# Affective Response Categories—Toward Personalized Reactions in Affect-Adaptive Tutoring Systems

**DOI:** 10.3389/frai.2022.873056

**Published:** 2022-05-17

**Authors:** Alina Schmitz-Hübsch, Sophie-Marie Stasch, Ron Becker, Sven Fuchs, Maria Wirzberger

**Affiliations:** ^1^Fraunhofer Institute for Communication, Information Processing and Ergonomics FKIE, Wachtberg, Germany; ^2^University of the Bundeswehr München, Neubiberg, Germany; ^3^University of Stuttgart, Stuttgart, Germany

**Keywords:** affect-adaptive systems, intelligent tutoring systems, affective user state, safety-critical systems, hierarchical cluster analysis

## Abstract

Affect-adaptive tutoring systems detect the current emotional state of the learner and are capable of adequately responding by adapting the learning experience. Adaptations could be employed to manipulate the emotional state in a direction favorable to the learning process; for example, contextual help can be offered to mitigate frustration, or lesson plans can be accelerated to avoid boredom. Safety-critical situations, in which wrong decisions and behaviors can have fatal consequences, may particularly benefit from affect-adaptive tutoring systems, because accounting for affecting responses during training may help develop coping strategies and improve resilience. Effective adaptation, however, can only be accomplished when knowing which emotions benefit high learning performance in such systems. The results of preliminary studies indicate interindividual differences in the relationship between emotion and performance that require consideration by an affect-adaptive system. To that end, this article introduces the concept of Affective Response Categories (ARCs) that can be used to categorize learners based on their emotion-performance relationship. In an experimental study, *N* = 50 subjects (33% female, 19–57 years, *M* = 32.75, *SD* = 9.8) performed a simulated airspace surveillance task. Emotional valence was detected using facial expression analysis, and pupil diameters were used to indicate emotional arousal. A cluster analysis was performed to group subjects into ARCs based on their individual correlations of valence and performance as well as arousal and performance. Three different clusters were identified, one of which showed no correlations between emotion and performance. The performance of subjects in the other two clusters benefitted from negative arousal and differed only in the valence-performance correlation, which was positive or negative. Based on the identified clusters, the initial ARC model was revised. We then discuss the resulting model, outline future research, and derive implications for the larger context of the field of adaptive tutoring systems. Furthermore, potential benefits of the proposed concept are discussed and ethical issues are identified and addressed.

## Introduction

“The extent to which emotional upsets can interfere with mental life is no news to teachers. Students who are anxious, angry, or depressed don't learn; people who are caught in these states do not take in information efficiently or deal with it, well.” (Goleman, 1995, p. 78)

The introductory Goleman quote succinctly presents what many studies suggest: Emotions influence learning success. This is not surprising since emotions influence motivational processes, attention allocation, and memory formation, which are important factors in learning success (Tyng et al., 2017). As *Intelligent Tutoring Systems* (ITSs) enable individualizing the learning experience as well as identifying and mitigating non-optimal learning conditions, these systems can be used to address the emotional state. ITSs that assess the learner's emotions and use this information to promote learning are called *Affective Tutoring Systems* (ATSs). De Vicente and Pain (1998) suggested that the learner state in ITSs should be diagnosed to induce a state of cognitive and emotional arousal so that the instruction is perceived as an interesting and engaging experience. D'Mello et al. (2008) similarly argue that learning environments monitoring emotions are motivating and relevant to the learner, but robust emotion detection is necessary to develop real-time, affect-sensitive tutoring systems.

An ATS can especially add value in training systems for safety-critical environments. Safety-critical systems are systems whose failure endangers human life, results in significant economic loss or property damage, or can cause environmental damage (Knight, 2002). Boy (1998) identifies four properties that characterize safety-critical systems: time pressure, high complexity, risk bearing, and dependence on human factors. Examples include aerospace systems, nuclear facilities, and military systems, especially weaponry. Due to the serious consequences of failures, it is important to support the student in developing coping strategies and improving resilience (Talker, 2021) on detected undesired emotional states. The goal of our research is to create an affect-adaptive training system for safety-critical work environments.

This investigation thus aims at answering the first research question: Which emotional states should be promoted to maintain performance in a training system for safety-critical work environments? Defining these emotional states is important to determine the direction of adaptations in a future affect-adaptive learning system. Cai and Lin (2011) searched for a sweet spot indicating the optimal emotional user state for the best performance in a driving simulator study. Previous research confirms the existence of sweet spots but also observed important individual differences in the locus of the sweet spot (Schmitz-Hübsch and Fuchs, 2020; Schmitz-Hübsch et al., 2021). This leads to the second question: How can affect-adaptive systems account for such interindividual differences? To address this challenge, our article introduces the concept of Affective Response Categories (ARC). This classification approach groups users into categories according to their emotion-performance relationship. A third research question addresses the feasibility of this classification concept: Can the initial concept of the ARCs be supported by empirical findings?

## Background

In this section, we provide a theoretical background regarding emotion, tutoring systems, and the relationship between emotion and performance. Thereafter, we describe the fundamental idea of the ARCs, the method used to investigate the research questions, the results of the analyses, and a discussion. The article closes with our conclusions drawn from this investigation.

### Classification of Emotion

**Figure 1:**
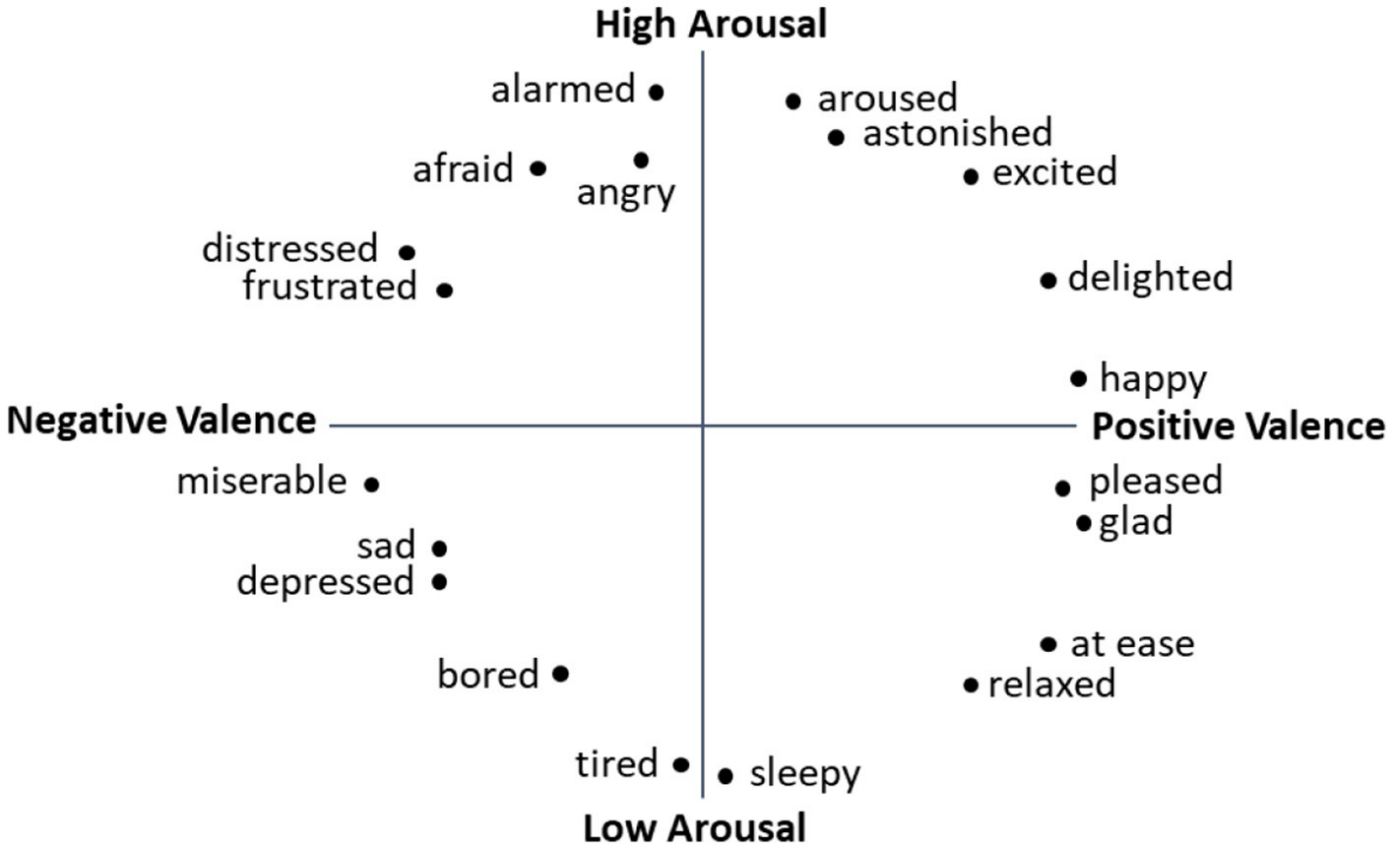
Circumplex Model of Affect (adapted from Russell, 1980).

A variety of emotion classification systems exists, for example categorical or dimensional classification. The dimensional approach originated in the work Wundt (1896), who formulated three pairs of opposites for categorizing emotions: pleasure and displeasure, strain and relaxation, and excitement and quiescence (or depression). In modern research approaches, the correlations between different emotions are investigated by analyzing the frequency of their co-occurrence (Schmidt-Atzert, 2019). A common dimensional definition involves Russell's Circumplex Model (Russell, 1980; see Figure 1), which employs two dimensions and represents their correlations in a matrix. Now titled “valence” and “arousal,” these dimensions form orthogonal axes of a coordinate system onto which any emotions can be plotted according to their levels of valence and arousal.

### Tutoring Systems and Emotion

Nkambou (2006) defines tutoring systems as computer-based learning systems that enable one-to-one interaction in teaching and learning. He refers to tutoring systems as intelligent when they present human-like tutoring capabilities, such as adjusting the content of the lesson to the students' characteristics and needs by analyzing their responses and behaviors. Another definition by Ma et al. (2014) notes that ITS are computer programs that provide individualized instruction by analyzing model learners' psychological states.

Petrovica et al. (2017) define ATS as an ITS adapting to a learner's emotional state with the aim to intervene when an emotional state negatively impacts knowledge acquisition and learning outcomes. According to them, a typical ATS consists of three components: one detects and classifies the student's emotional state. Another uses data about the current emotional state and the tutoring situation to generate appropriate responses and adaptations. The third is an emotion expression module, which enables the ATS to express emotions depending on students' emotions and actions in the form of a virtual tutor or pedagogical agent (Petrovica et al., 2017).

User ethics represent a challenge in affect-adaptive systems that capture and possibly manipulate the emotional state. Fairclough (2009), for example, describes issues of privacy and user autonomy that should be considered in the design and implementation of such systems. The present article therefore discusses issues of user ethics regarding the appropriate use of the proposed concept and system in the context of current literature.

Forbes-Riley et al. (2008) modeled learning success in a language-based physics learning system with the goal of developing an affect-adaptive learning system. Various parameters, such as speech recognition quality, dialogue efficiency, and affective user state, predicted learning success in a multiple linear regression. The results showed that models including affect significantly outperformed models without it, indicating the relevance of emotional state to learning success. The research of Nkambou (2006) is closer to an operational system, who demonstrated an ATS aiming to capture the emotions expressed by the learner *via* facial expression analysis and to control them. This system was intended to provide flexible tutoring, contextual help, and explanations during the learning process. It utilized a “pedagogical loop” which resembles the three components described by Petrovica et al. (2017).

Arroyo et al. (2009) used various sensors to capture the emotional user state in an ITS for math training. The system was designed to detect the students' emotional state and provide emotional support. In addition to emotional facial expression, it analyzed skin conductance, sitting posture, and mouse-click pressure. The authors recommend further development of emotional models to predict desired and undesired learning states for future work, which our contribution aims to address. The identification of desired and undesired emotional states is especially important in training systems for safety-critical environments.

### Relationship Between Emotion and Performance

In cognitive psychology, the affect-as-information hypothesis by Gasper and Clore (2002) highlights that emotions influence cognitive functions of memory, judgment, decision-making, and information processing. According to Clore and Huntsinger (2007), negative emotions lead to local, item-specific, and stimulus-based information processing, whereas positive emotions promote top-down and theory-based processing (Clore and Bar-Anan, 2007). Baddeley (1972) found that performance is impaired in dangerous situations, and Shields et al. (2016) showed that anxiety reduced performance relative to a neutral emotional state, while anger does not reduce performance in this way. Furthermore, Grossman and Christensen (2007) postulated that performance in military environments is increased when fear is low.

#### Emotion and Performance in Human-Machine Systems

On the one hand, regarding studies about human-machine systems, negative emotions seem to decrease performance. Kontogiannis (1996) found that operators who were stressed, angry, or frustrated could not reach their optimal performance level. Furthermore, Wells-Parker et al. (2002) showed that negative emotions associated with high arousal levels, such as anger, also impair driving performance. On the other hand, Panganiban (2011) showed that operators with high trait anxiety had a better performance; however, this effect could not be shown for state anxiety. James and Nahl (2000) showed that a positive mood is a favorable prerequisite for safe driving, but Zimasa et al. (2017) found no positive effect of happiness on reaction times concerning road hazard recognition.

In an ITS, Woolf et al. (2009) found that emotional states of negative valence and high levels of arousal are unproductive and therefore undesirable. Following a review of literature regarding emotion regulation, Malekzadeh et al. (2015) similarly concluded that a positive emotional state has several benefits for learners, such as promoting greater cognitive flexibility and enabling discovering new ideas and possibilities. Negative emotional states, such as boredom and frustration, were related to lower use of self-regulation and cognitive learning strategies as well as an increase in unfocused and disruptive behavior during learning. In contrast, Picard (2000) emphasized the rewarding “aha effect” after experiencing frustration or anxiety due to increased task difficulty. If one can overcome these negative emotions, frustration can be helpful by becoming a source of motivation. These contradicting statements indicate that the relationship between emotion and learning performance is rather complex and needs further exploration.

#### Interindividual Differences in the Relationship Between Emotion and Performance

In a previous investigation (*N* = 22, 17–52 years, *M* = 30.9, *SD* = 9.7) of emotional state diagnosis, the relationships between positive or negative valence and performance were analyzed on an individual level (Schmitz-Hübsch and Fuchs, 2020). The observed experimental task originated from a command-and-control system representing a safety-critical environment. The facial expression classification software Emotient FACET (Littlewort et al., 2011) assessed emotional valence in two different classifiers (positive and negative valence), whereas task performance was assessed by counting performance decrements that were triggered whenever tasks were not completed in time. Individual regressions were calculated per subject.

37% of subjects showed significant correlations of positive valence and performance, but their direction varied as 14% showed high performance and 23% showed low performance associated with positive valence. Similar results were found for the correlation between negative valence and performance, where 18% of subjects showed negative valence associated with high performance while 27% showed low performance. These results indicated interindividual differences in the relationship between emotional valence and performance.

In a further investigation, data from two additional experiments were examined (*N* = 24, 19–48 years, *M* = 32.0, *SD* = 7.2 and *N* = 16, 22–49 years, *M* = 32.3, *SD* = 8.7; Schmitz-Hübsch et al., 2021). In addition to positive and negative valence, another Emotient FACET classifier was added, namely neutral valence. Following the Circumplex Model of Affect (Russell, 1980), emotional arousal was also included in the analysis to more comprehensively portray the emotional state of the user. The arousal dimension was operationalized using three physiological indicators: heart rate, heart rate variability, and pupil diameter (Schmitz-Hübsch et al., 2021).

Consistent with Cai and Lin (2011), it was hypothesized that a high percentage of subjects would show high performance when positive and negative valence were low and neutral valence was high. Following Staal (2004), who found that highly arousing states decrease the operators' performance, low performance was expected to be associated with high arousal, meaning when heart rate and pupil diameter are high and heart rate variability is low.

The majority subjects showed correlations between valence and performance and would therefore benefit from an affect-adaptive system. Detailed results exhibited interindividual differences in valence. In both experiments, statistical trends indicated that low positive and low negative valence are associated with high performance; however, in some cases, performance was better with high positive or negative valence, while no correlation between valence and performance was apparent in others. As expected, neutral valence tended to be associated with high performance, but interindividual differences were again present.

High arousal was overall associated with lower performance in both experiments. In particular, high pupil diameter was associated with low performance in most subjects, while high heart rate was associated with low performance in most cases. For heart rate variability, however, interindividual differences in the direction of the association were present.

### Hypotheses: Affective Response Categories

The observed interindividual differences pose a major challenge for the development of affect-adaptive systems. The goal of an affect-adaptive safety-critical system is to respond to critical emotional states associated with low performance; however, non-critical emotional states associated with high performance do not require adaptation. Our previous research indicates that a universal definition of these states on the circumplex plane is not possible, as our results suggest different directions in the emotion-performance relationship. One person could have a non-critical state in the positive valence area and a critical state in the negative area, whereas the areas could be reversed for another person. An affect-adaptive system should be able to interpret these states individually.

Furthermore, the selection of adaptations must be based on the individual relationship between emotion and performance. Although data from previously described investigations showed a tendency toward the neutral emotional user state as the sweet spot of performance, a system that promotes the neutral state may impair subjects who show the highest performance in positive or negative states. Affect-adaptive systems should hence consider interindividual differences, for example, by assigning users to specific classes that differ in emotion-performance relationships. The previous investigations indicate that some participants show greater similarities in emotional response patterns regarding performance than others. These similarities in individual responses can be used to group individuals with the matching emotional response pattern, which would allow for easier classification in the envisioned affect-adaptive learning system.

**Figure 2:**
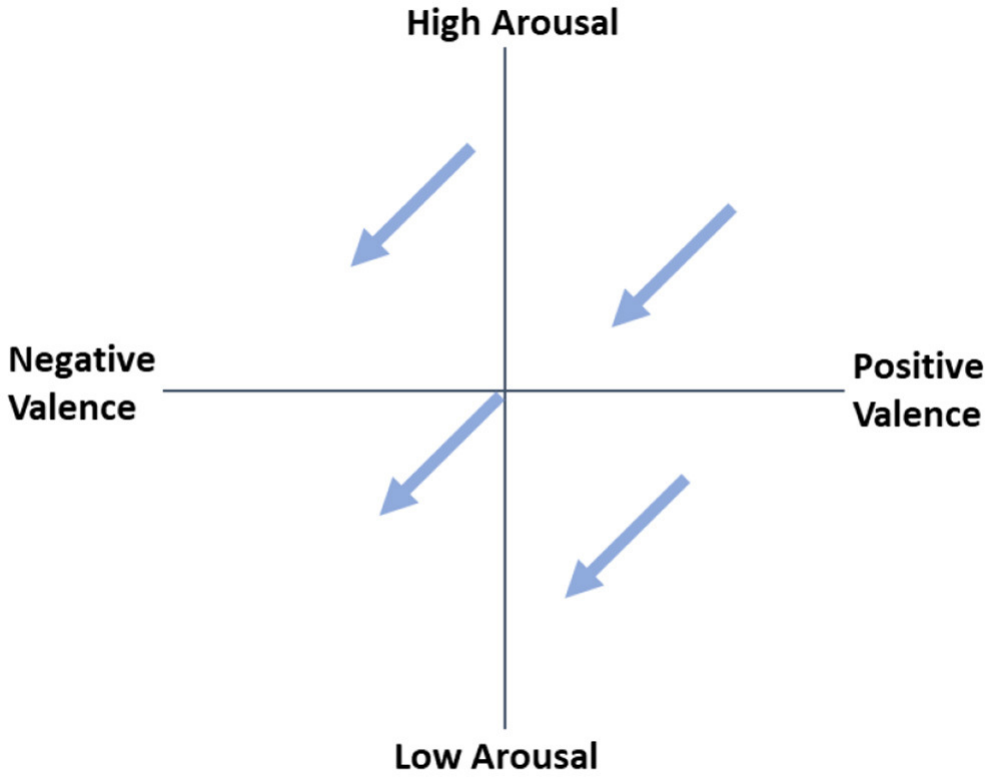
Adaptation direction for a negative relationship between both valence and arousal and performance with different origins.

To this end, we propose assigning affect-adaptive system users to ARCs that differ in the relationship between emotion and performance. These categories represent the direction in which adaptation mechanisms modulate valence and arousal on the Circumplex Model of Affect (Russell, 1980) to optimize individual performance. ARCs can be illustrated using arrows between valence and high performance and between arousal and high performance. The origin of the arrow represents the current emotional state of the user and may vary over time. Figure 2 illustrates the optimal adaptation direction for a negative relationship between both valence and arousal and performance in combination with different starting points.

**Figure 3:**
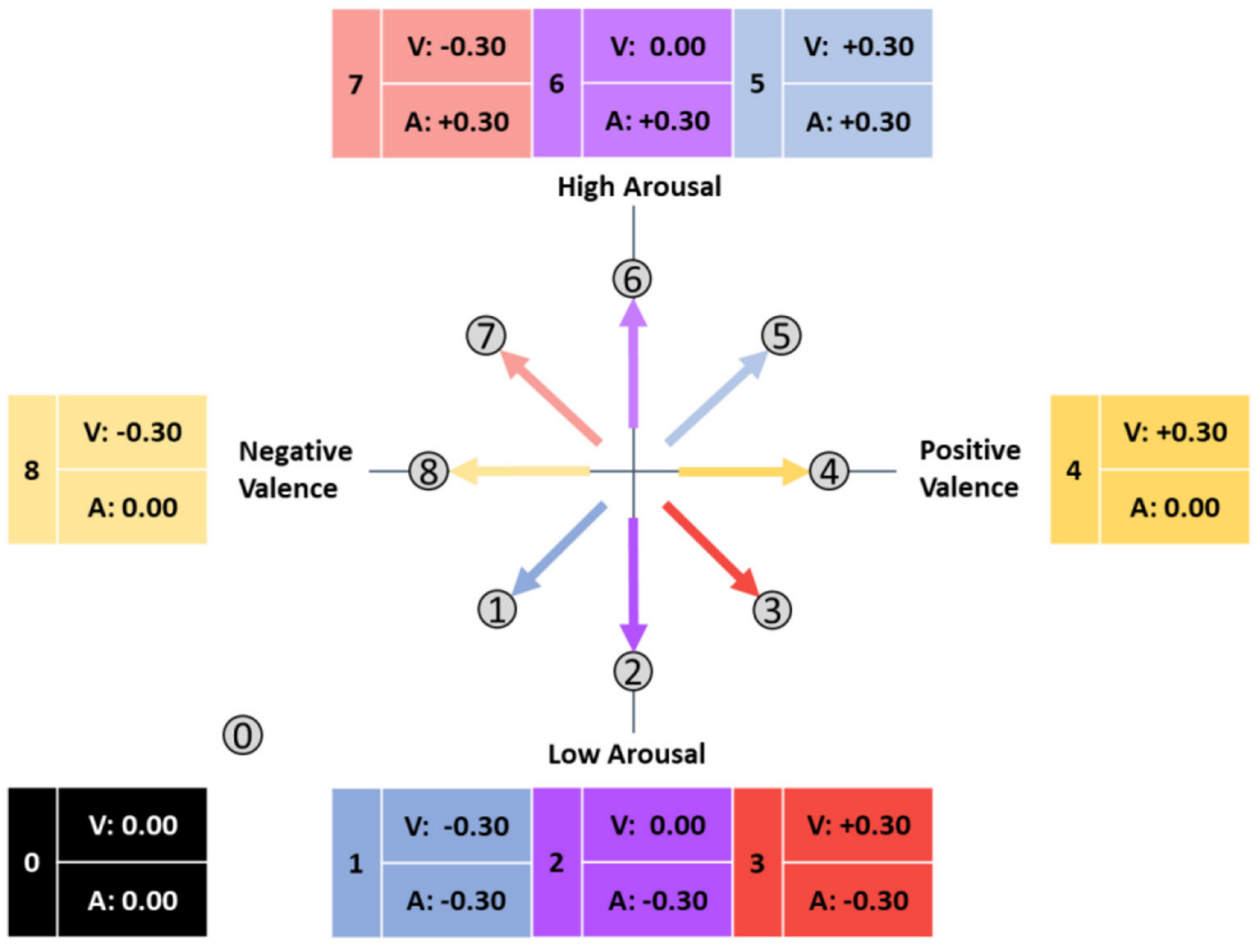
Possible affective response categories in the circumplex model of affect (adapted from Russell, 1980).

If an ARC points left, it indicates a negative correlation between valence and performance; if it points right, it indicates a positive correlation. The lateral alignment similarly reflects the relationship between arousal and performance. Later on, the direction of an arrow will indicate the direction of the emotional adaptation. Three directions are possible for the correlations between the two emotion dimensions and performance: positive correlation, negative correlation, and no correlation, which allows for eight possible ARC directions (see Figure 3, Table 1). For comparability, we illustrated all ARCs as starting form a neutral origin, although any point could serve as the starting point depending on the current emotional state of the individual. The following table illustrates the adaption goals for each ARC.

**Table 1:** Adaption goals for arousal and valence depending on the ARC.

**ARC**	**Arousal should be..**.	**Valence should be…**
1	Decreased	Decreased
2	Decreased	—No adaption needed—
3	Decreased	Increased
4	—No adaption needed—	Increased
5	Increased	Increased
6	Increased	—No adaption needed—
7	Increased	Decreased
8	—No adaption needed—	Decreased
0	—No correlation between emotions and performance at all—	

Following a meta-analysis by Zaehringer et al. (2020) that found small to medium psychophysiological effects of negative emotions, and Lundkvist et al. (2021) who found a medium effect in the relationship of emotion and performance, the magnitude of the assumed correlations was estimated to be approximately the absolute value of 0.3, corresponding to a medium effect according to Cohen (1988). The expected mean correlation coefficients between performance and both valence (V) and arousal (A) are plotted around the circumplex plane in Figure 3. A precise statement about the target area of adaptation cannot be derived from this concept and visualization, and the exact angle of adaptation cannot be described by the arrow.

**Figure 4:**
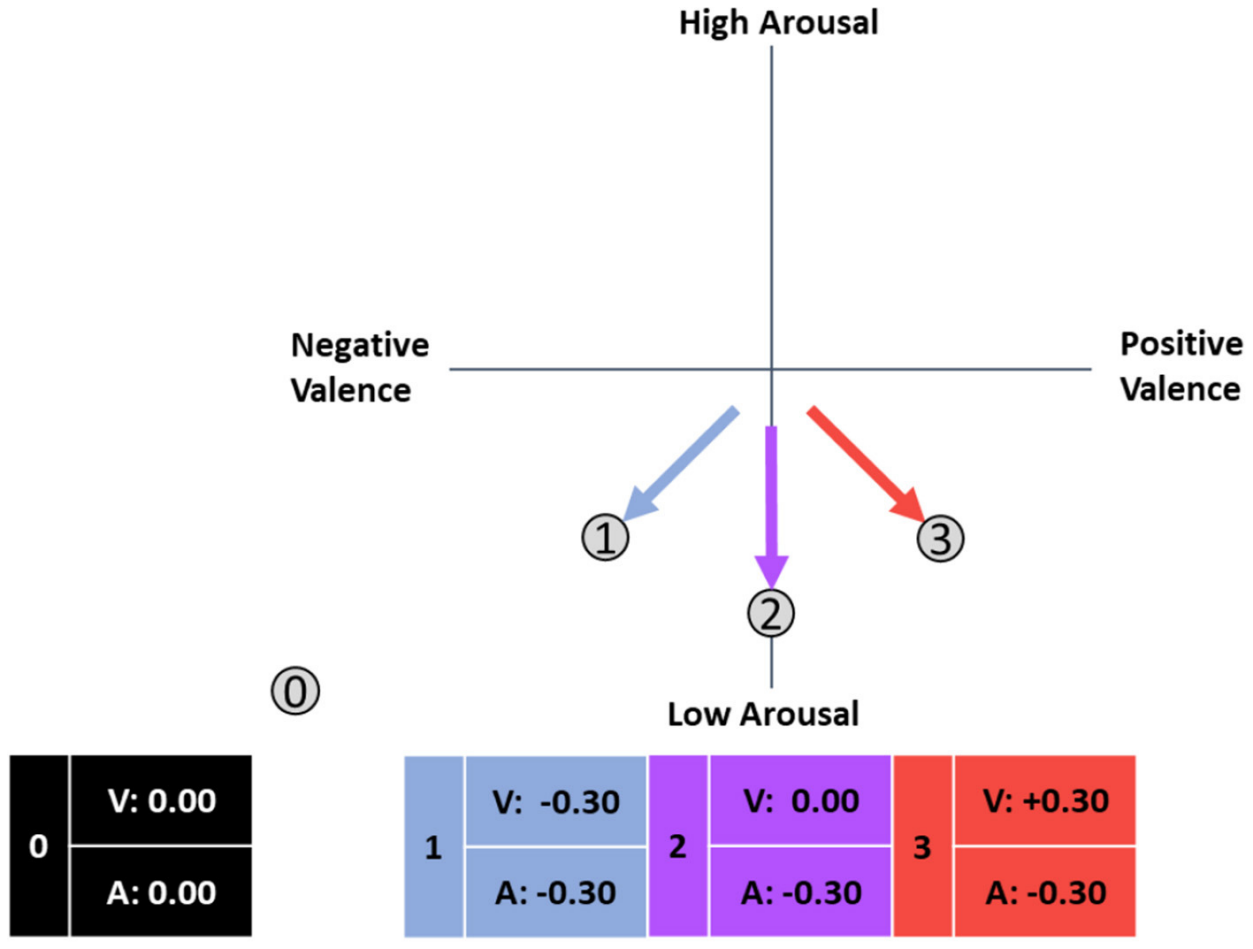
Proposed framework of affective response categories in safety-critical systems in the circumplex model of affect (adapted from Russell, 1980).

In our investigation of data from a training system for safety-critical tasks, we found a negative association between arousal and performance, where low arousal was associated with high performance (Schmitz-Hübsch et al., 2021). Furthermore, the safety-critical task context and potentially fatal consequences of a mistake might result in a certain stress level, which in turn is associated with an increased arousal level. Further increasing the arousal level would overshoot the optimal level proposed by the Yerkes-Dodson-Law (Yerkes and Dodson, 1908). Based on these results, we propose that affect-adaptive systems for such task environments should focus on the discrimination of ARCs 0 through 3 (see Figure 4):

0. No relationship between emotion and performance.
1. High performance is associated with negative valence and low arousal.
2. High performance is associated with neutral valence and low arousal.
3. High performance is associated with positive valence and low arousal.

We hypothesize that only ARCs 0 through 3 occur in safety-critical simulations. An empirical evaluation of the proposed ARC concept is provided from Section Methods onwards.

### Ethical Considerations

Ethical considerations are becoming increasingly important in the development of artificial intelligence (AI) systems (Hagendorff, 2020). The ethical use of affect-adaptive tutoring systems in safety-critical environments is crucial, because emotions majorly influence cognitive processes such as decision making (Andrade and Ariely, 2009), moral judgement (Horberg et al., 2011), attention (Phelps et al., 2006), perception (Zadra and Clore, 2011), or memory (Christianson, 2014). The planned system has two core functions: recognizing the emotional user state and manipulating maladaptive affective states toward the emotional sweet spot. The intertwinement of emotion, cognition, and behavior, requires a clear ethical framework and division of responsibility between human and AI.

Ong (2021) developed an extensive framework for guiding the development of affect-aware AI based on two pillars, namely *provable benefice* and *responsible stewardship*. The former addresses the responsibility of the system developer, while the latter refers to the responsibility of the future operator, the so-called *emoter*. In the following section, each guideline is applied to the current development state of our affect-aware tutoring system. Since the system is currently in the early development phase, this section focuses on the subprinciples underlying provable benefice, for which the developer is responsible.

The core function of an affect-adaptive tutoring system regards manipulating maladaptive emotional user states toward the emotional sweet-spot, thereby maintaining a high performance level of the user in safety-critical systems.

Steinert and Friedrich (2020) discuss ethical issues related to affective brain-computer interfaces (BCIs), which share the principle of influencing the user's emotion while interacting with a technical system. Firstly, the authors note that recordings about the emotional user state regard sensitive personal data and underlie the principles of data protection. The informed consent of using the system is a prerequisite for every use, and future operators need to ensure that the emoter is always aware of and agrees to the monitoring of their emotion. Moreover, a system must preserve the mental integrity of the person, meaning that the user has control over their mental states and the recorded data. An explainable intelligent tutoring system related to the concept of explainable AI (Hagras, 2018; Hoffman et al., 2018; Holzinger, 2018) offers a solution to achieve this goal. Doran et al. (2017) extend the concept of an explainable AI to a truly explainable AI by integrating a reasoning engine to an AI system. The reasoning engine explains the decision-making process of a model to the user by utilizing understandable features, which overcomes the uncertainty regarding how the system has reached its decision for emotional adaptation. In an affect-adaptive system, the user should consequently be aware of the processes related to the emotion recognition and sweet-spot adaptation. This concern relates to the issue of responsibility of emotion regulation and associated actions of the emoter. The responsibility for one's own emotion regulation should not be transferred to the machine, because the emoter is responsible for their strategic planning along with the final decision making (Butlewski, 2017), which requires consideration by the developers of an affect-adaptive tutoring system during the development process. About 32% of participants showed a sweet spot within a negative valence range; however, the adaptation of emotion toward a negative sweet spot contradicts the notion of Cowie (2015), as the interaction between human and machines should be more likely to generate positive affect and less likely to generate negative affect. The benefit of improving users' performance in the learning environment, however, serves the subprinciple of provable benefice (Ong, 2021).

Several ethical guidelines emphasize the benefit of the system in the development of affective AI (Floridi et al., 2018; Jobin et al., 2019; Siau and Wang, 2020). Although employing affect-adaptive tutoring systems entails some limitations regarding emotion recognition and adapting emotional user states, the benefits of promoting learning and maintaining a high performance outweigh their costs. Technical developments regarding explainable and responsible AI as well as context-sensitive emotion recognition algorithms may further enhance the benefits while keeping the associated costs at a low level.

## Methods

In order to empirically validate the proposed concept, we conducted a laboratory experiment. The following section describes the sample, study design, materials used and the experimental task.

### Participants

Data were collected from *N* = 50 subjects (33% female, 19–57 years, *M* = 32.75, *SD* = 9.8). All participants were employees of the Fraunhofer Institute for Communication, Information Processing and Ergonomics (FKIE) and were invited *via* e-mail. Participation was not compensated, but for motivational purposes, the three best-performing subjects earned an online shopping voucher. The study was approved by the ethics committee of the University of Chemnitz.

### Design

Three variables were recorded during the experimental task: emotional valence, emotional arousal, and performance. A subdivision into dependent and independent variables is not possible since the conducted correlation procedure is part of interdependency analysis.

Task difficulty was varied in a within-subjects design. All subjects completed 12 scenarios that differed in the number of tasks, and the scenarios were presented in a randomized order to avoid sequence effects. After each scenario, affective self-report questionnaires were presented along with an assessment of the Big Five personality factors. Both this information was not used in the present investigation.

To categorize subjects according to their emotion-performance relationships, we performed a hierarchical cluster analysis with the Pearson correlation coefficients.

### Materials

To capture emotional valence, continuous user emotional state classification based on facial expressions (Emotient FACET; Littlewort et al., 2011) was employed. A Logitech C920 webcam recorded videos of the subjects' faces with a frame rate of 25 Hz. The camera was placed on the top center of the monitor. Emotional arousal, which is physiologically associated with increased pupil diameter (Bradley et al., 2008), was recorded using a Tobii Pro Spectrum 300 Hz Eye Tracker. Further arousal measures (heart rate and heart rate variability) were recorded but not further analyzed because of the higher inconsistency in their relationship with performance compared to pupil dilation (Schmitz-Hübsch et al., 2021). Performance was assessed *via* a performance score that considered the priority of the tasks, accuracy, and response time (Becker et al., 2021). To determine the Big Five personality factors, the NEO-FFI questionnaire was used.

### Experimental Task

The experimental testbed was implemented based on the Rich and Adaptable Test Environment (RATE; Becker et al., 2021), which is a software toolbox that allows the modular, scalable, and efficient development of research testbeds based on a modern software architecture. Selecting, adjusting, or adding RATE modules enables adapting the result to a wide variety of requirement situations. In this study, a configuration called RATE for Command and Control (C2) was used, which is a simulation-based training environment comprising an airspace surveillance task that mimics cognitive demands and situation awareness requirements found in military C2 environments. This type of simulation is often utilized in training to improve performance in safety-critical situations. Users of this gamified simulation train the cognitive skills required, for example, in drone defense, as operators requiring situational awareness, control centers in power plants, or traffic control centers. Orasanu and Backer (1996) recommend skill training because it facilitates better performance in real-world, safety-critical situations under stress. For this reason, we selected an airspace surveillance task as a simplified representation of a real-world, safety-critical system and presented different scenarios with varying task demands.

**Figure 5:**
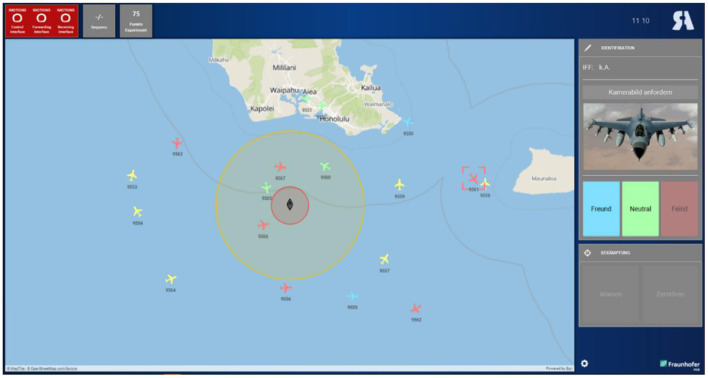
Screenshot of the experimental testbed (based on RATE for C2).

The experimental C2 task has the goal to protect the trainee's ship (see Figure 5, center) from enemy aircraft, and is comprised of three subtasks:

1. The *identification* of unknown aircraft (yellow) is based on the identification friend or foe (IFF) code and a camera image. Aircraft with IFF code 4 are to be identified as friendly by clicking on the blue button. If the IFF code is unknown, a neutral (commercial airliners) or hostile (fighter jets) identity is assigned based on the camera image.
2. Enemy aircraft approaching the user's ship represent a potential threat. Upon entering the outer perimeter around the user's ship, hostile contacts must be *warned*.
3. Hostile contacts that proceed into weapon range (inner perimeter) must be *engaged* (buttons at the bottom right).

The score was displayed in the upper-left corner of the screen (see Figure 5), enabling participants to assess their performance at all times.

Each scenario consisted of three attack waves, each 70 s long. There were 6, 12, 18, or 24 contacts in one wave to cover a broad spectrum of difficulty levels, adding up to three scenarios, each with 18, 36, 54, or 72 contacts in total. The number of tracks, time, and order of appearance were based on the Warship Commander Task (St. John, 2004). After each wave, all contacts were removed.

### Procedure

The experiment was carried out during the Covid-19 pandemic, which required strict sanitary measures and compliance with all applicable government regulations. First, subjects signed the privacy statement and informed consent document. A demographic questionnaire was subsequently presented before the sensor system was donned and calibrated. To calibrate the facial expression analysis and detect distinct facial features like glasses or a beard, participants were asked to look at a blank screen with a neutral expression for 6 s. For eye tracking calibration, we considered a five-point calibration sufficient since we only analyzed pupil diameter. A written instruction of the task followed. In a training scenario, participants familiarized themselves with the experimental task and were encouraged to ask questions. When subjects felt confident with the task, the trial began, which comprised 12 scenarios with subsequent affective self-report questionnaires. During the trial, talking was discouraged to avoid confounding emotion detection. After a short debriefing, the subjects were dismissed.

### Scoring

Before analyzing the data and performing the cluster analysis, it was necessary to prepare the data using several steps.

First, we explored the collected data and discovered ceiling effects in the performance metric in both the 6- and 12-track waves for most participants. Since ceiling effects could have obscured the relationship between emotion and performance, we excluded the affected scenarios despite the loss of data. In all other scenarios, the first wave was eliminated because the performance was not comparable to the subsequent waves. This effect was caused by the sudden appearance of many tracks at the beginning of the scenario, representing a characteristic of the WCT based on which the scenarios were modeled. Once all contacts of a wave were processed, the performance reached a plateau, which was also removed from the data.

#### Valence

The Emotient FACET engine offers three non-dependent scales for classifying valence: positive, negative, and neutral valence. Mapping these scales to the single valence dimension of the Circumplex Model of Affect (Russell, 1980) required merging them into one scale. For this purpose, a conversion was performed using a regression equation as detailed in Schmitz-Hübsch and Becker (In Press). The equation was developed by using FACET to analyze about 325,000 valence-labeled images from the AffectNet database (Mollahosseini et al., 2017). In a multiple regression analysis, FACET classifier values predicted database valence and explained 38% of the variance. Outliers were subsequently excluded using the 1.5^*^interquartile range method, and the data were normalized within a person (min-max method).

#### Arousal

Outliers were excluded using the 1.5^*^interquartile range method. Due to differences between subjects regarding pupil diameter and their response, they were normalized per subject (min-max method) and thus mapped onto the arousal dimension of the Circumplex Model of Affect (Russell, 1980).

#### Performance

RATE assesses performance using a point score that considers correct and timely task completion (Becker et al., 2021). Outliers were excluded using the 1.5^*^interquartile range method, and the performance was then normalized per subject using the min-max method.

#### Calculation of Time Windows

The performance data were assessed and recorded based on task events, where FACET assessed emotional valence at 30 Hz and pupil diameter was sampled at 120 Hz. To unify measures of different sample rates, the individual sample values of valence, arousal, and performance were averaged across sequential time windows. Based on literature findings (Katsis et al., 2008; Hosler et al., 2021), a time window of 10 s was determined to be appropriate.

## Results

A descriptive analysis gained initial insights into the data structure. For this purpose, the means and standard deviations of normalized valence, arousal, and performance were calculated for each of the four difficulty levels.

To investigate the direction and magnitude of the respective emotion-performance relationship, we calculated two product-moment correlations for each subject. The performance was correlated with valence (V^*^P) and with arousal (A^*^P). A hierarchical cluster analysis grouped subjects by these two correlation coefficients, and analysis was performed using the *cluster* package (Maechler et al., 2021) in R (R Core Team, 2021). The selection of the number of clusters was not predefined in the analysis but based on the silhouette index (Rousseeuw, 1987). Euclidean distance served as the distance measure and Ward as the clustering algorithm. To examine cluster properties, the mean of the correlation coefficients (V^*^P/A^*^P) of the subjects in a cluster was determined.

### Descriptive Analysis

Table 2 presents the means and standard deviations of performance, valence, and arousal of the normalized data for the two difficulty levels across all subjects. The standard deviations of performance decreased with higher difficulty, the valence means and standard deviations were constant across difficulty levels, and the arousal means increased with higher difficulty.

**Table 2:** Descriptive analysis of performance, valence, and arousal.

**Difficulty level**	**Performance**		**Valence**		**Arousal**	
	**Mean**	**SD**	**Mean**	**SD**	**Mean**	**SD**
3	0.62	0.20	0.33	0.17	0.46	0.13
4	0.53	0.17	0.33	0.17	0.48	0.13

**Figure 6:**
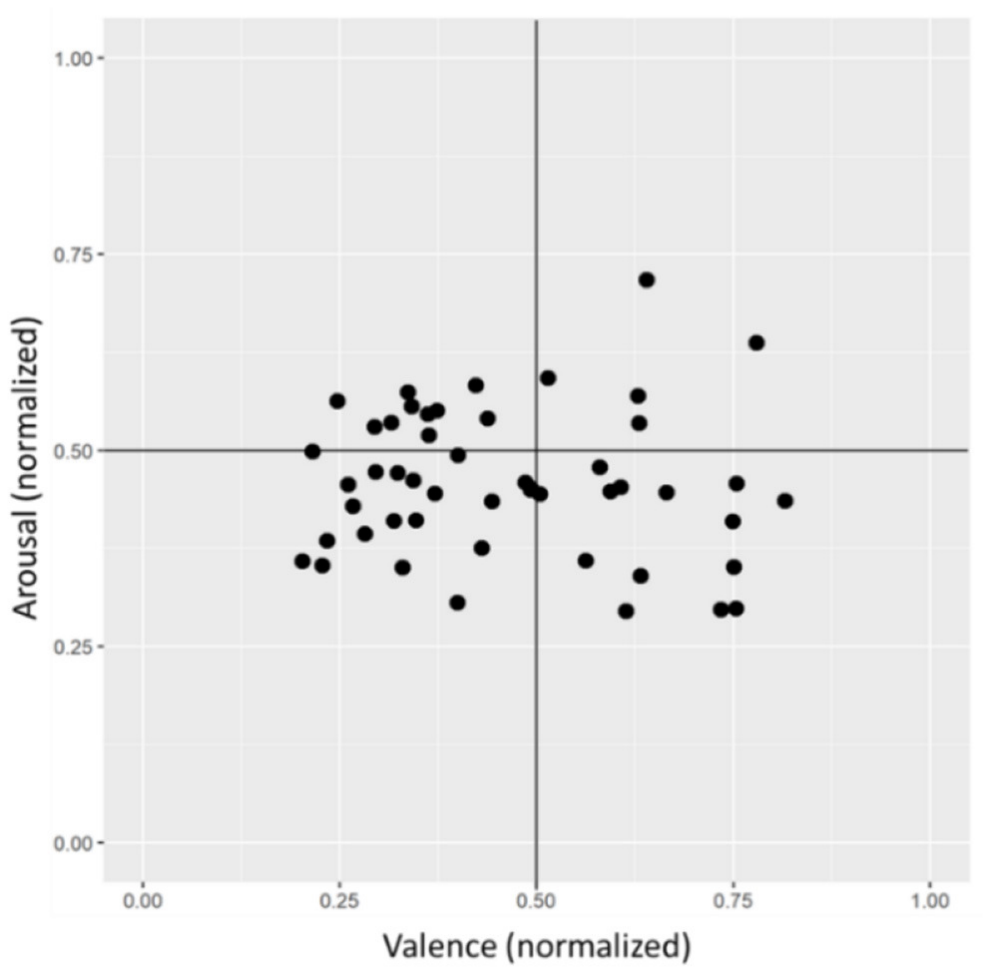
Scatterplot of the average general emotion of all subjects across the experiment.

The standard deviations of valence appeared high compared to their means, so individual differences in the subjects' average emotion were further explored in a scatterplot (see Figure 6), which shows the mean values of all subjects regarding valence and arousal. The general emotion in the experiment strongly differed between subjects, and high interindividual variance is apparent in both dimensions, especially valence.

### Cluster Analysis

**Figure 7:**
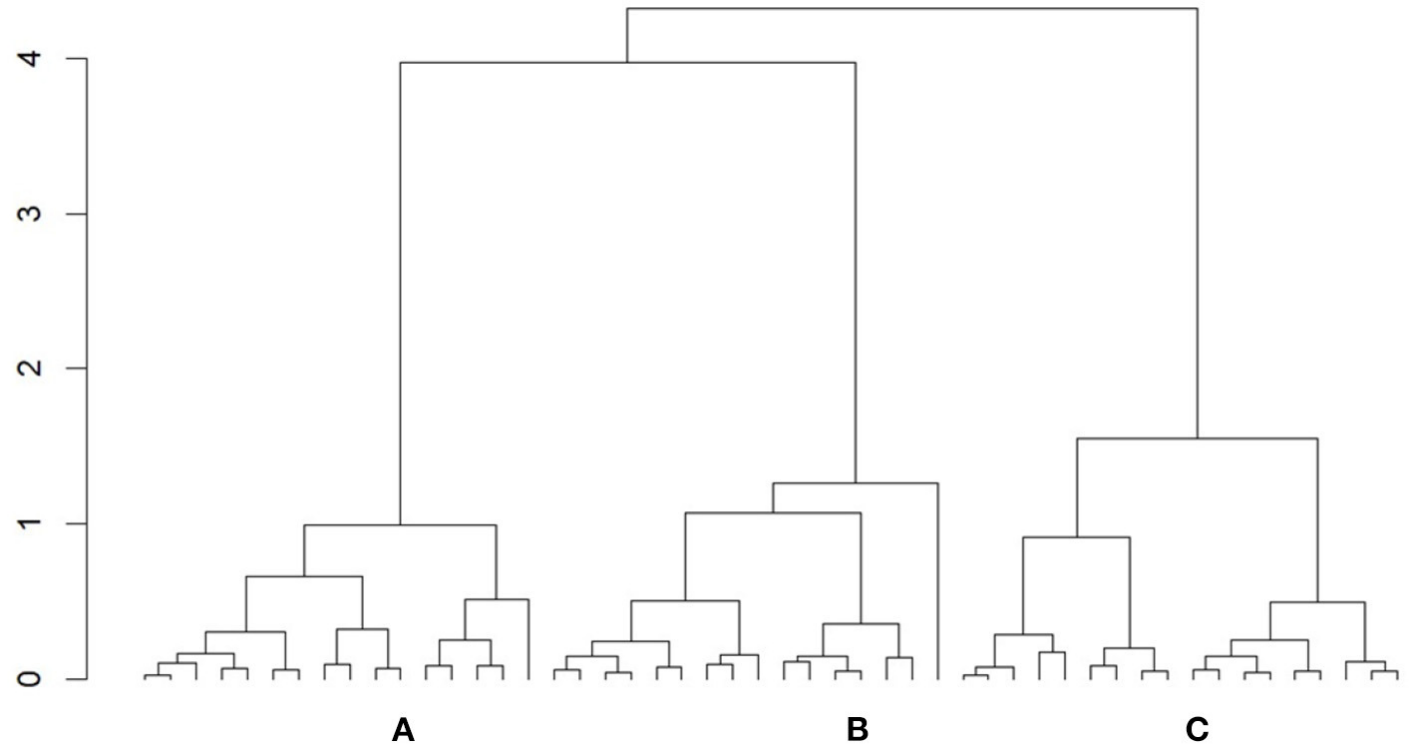
Dendrogram of the cluster analysis with the three cluster **(A–C)**.

The result of the cluster analysis can be visualized in a dendrogram (see Figure 7), where subjects with similar correlation coefficients are grouped together. The greater the similarity, the shorter the vertical line or height. Following a first visual impression, three or four clusters could be selected. To determine the optimal number of clusters, we additionally calculated the silhouette index, which validates the clustering performance (Rousseeuw, 1987). The values range between −1 and 1, and the maximum value indicates the ideal number of clusters. An average index of 0.25 or lower indicates that there is no structure present, while a weak structure is obtained between 0.25 and 0.5, a medium structure between 0.5 and 0.7, and a strong structure higher than 0.7 (Kaufman and Rousseeuw, 2009). While the index value for three clusters amounts to 0.376, it was only 0.294 for four clusters. A higher or lower number of clusters was not beneficial according to the silhouette index. In addition, the four-cluster alternative contains subjects with negative silhouette indices, which indicates that subjects may be in the wrong cluster. Three clusters consequently best divide the data (highest silhouette index with no single negative index value), and the index confirms a (weak) structure. The frequency distribution appears even, where 32% of subjects belong to clusters A and B, respectively, while 36% belong to cluster C.

**Figure 8:**
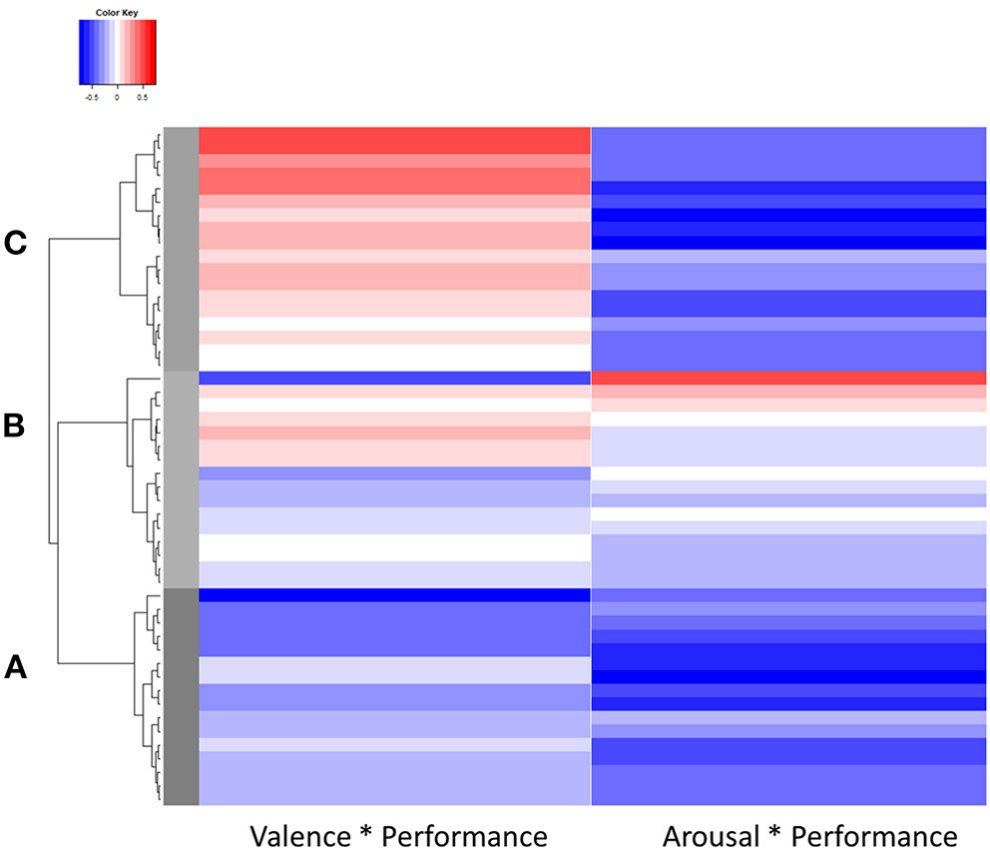
Heat map of the cluster analysis.

To explore the clusters in more detail, a heat map was created (see Figure 8) containing both correlation coefficients per subject in the respective cluster. A negative correlation is coded blue, a positive correlation red, and no correlation white. Gray bars illustrate the boundaries of the clusters. In the heat map, correlations between valence and performance appear to vary in cluster A, from strongly positive to weakly positive to no correlation. Cluster B shows weak positive and weak negative while cluster C shows exclusively negative correlations. The heat map further illustrates consistent negative correlations between arousal and performance in clusters A and C. In cluster B, however, both weak positive and weak negative correlations are represented.

Prior to further processing, the individual clusters were checked for normal distributions to enable subsequent calculation of inferential statistics. Cluster A and B each contained one outlier that was removed. To now assign clusters A, B, and C to the originally hypothesized ARCs 0 to 8 (cf. Figure 3), of which we expected only ARCs 0 to 3 to occur (cf. Figure 4), the mean values of the correlation coefficients within a cluster were calculated. The results, expected values, and a preliminary assignment to the ARCs are shown in Table 3.

**Table 3:** Assignment of clusters to hypothesized ARCs based on mean of the correlation coefficients within the clusters.

**Cluster**	**ARC**	**Arousal should be..**.	**Valence should be…**	**r**_**Valence, Performance**_		**r**_**Arousal, Performance**_	
				**Expected**	**Observed**	**Expected**	**Observed**
A	1	Decreased	Decreased	−0.30	−0.24	−0.30	−0.45
B	0	—No correlation between emotions and performance—		0	−0.03	0	−0.08
C	3	Decreased	Increased	0.30	0.19	−0.30	−0.44

**Figure 9:**
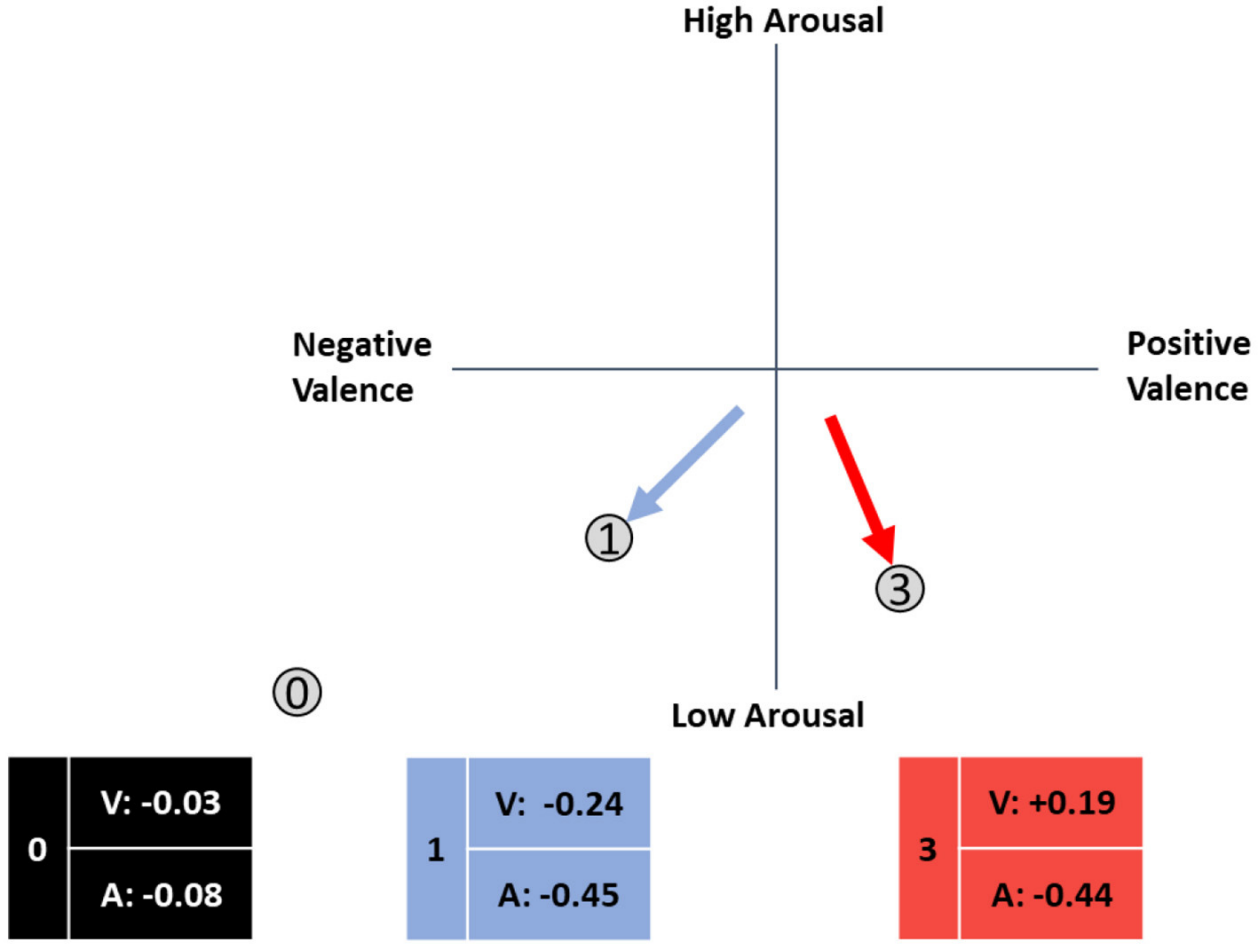
Average correlation coefficients in ARCs based on cluster analysis in the circumplex model of affect (adapted from Russell, 1980).

Figure 9 shows the mean correlation coefficients of the clusters assigned to ARCs in the Circumplex Model of Affect (adapted from Russell, 1980).

- Cluster A shows a negative relationship between performance and valence and arousal and thus corresponds to ARC 1.
- No association between emotion and performance was found in the averages of cluster B, corresponding to ARC 0.
- Cluster C combines positive valence-performance relationships of different magnitudes with a strong negative arousal-performance relationship, and thus corresponds neither to ARC 2 nor to ARC 3 in an obvious. The observed value of 0.19 lies close to the middle of the expected values for ARC 2 (0) and ARC 3 (0.3), which indicates that these two clusters may have merged into one. We assigned this merged cluster to ARC 3 since, as hypothesized for this category, there was a positive correlation for valence with at least a small effect according to Cohen (1988), even though it was weaker than expected. The associated arrow was shifted downwards.

To avoid overfitting the model by adjusting it to the empirical findings, we validated the clusters found. Six one-sample *t*-tests, one for each ARC and the correlation coefficients of valence and arousal, were used to test whether the mean values of the observed clusters correspond to the expected values of the ARCs or whether there are significant differences. To counteract the problem of multiple comparisons, we used the Holm test procedure (Holm, 1979). Table 4 shows the results of this investigation, which includes differences found for all ARCs. The mean observed arousal correlation coefficients of every ARC were higher than the expected effect. Regarding valence, there was no difference present for ARC 1; however, the observed correlation coefficients were lower than expected in ARC 3.

**Table 4:** Results of one-sample *t*-tests.

	Valence*Performance		Arousal*Performance	
**ARC**	**Results *t*-test**	**Difference**	**Results *t*-test**	**Difference**
0	*t*_(14)_ = −0.99, *p* = 0.336	Not significant	*t*_(14)_ = −2.79, *p* = 0.043	Significant
1	*t*_(14)_ = 2.02, *p* = 0.125	Not significant	*t*_(14)_ = −4.80, *p* = 0.002	Significant
3	*t*_(17)_ = −2.97, *p* = 0.034	Significant	*t*_(17)_ = −4.49, *p* < 0.002	Significant

## Discussion

To develop an affect-adaptive training system for safety-critical work environments, we investigated the optimal emotional user state for best possible performance. Previous studies showed important individual differences in the locus of this sweet spot (Schmitz-Hübsch and Fuchs, 2020; Schmitz-Hübsch et al., 2021). These differences in the emotion-performance relationship pose a challenge for designing affect-adaptive systems since fully individualized adaptation mechanisms would require personal settings. Cost and effort could be greatly reduced if it were possible to group users into different categories based on their emotion-performance relationship. We assumed that users of affect-adaptive systems can be assigned to ARCs, based on the Circumplex Model of Affect (Russell, 1980), which differentiate this relationship. We proposed that affect-adaptive systems should focus on the discrimination of four ARCs (ARC 0–3). The ARC concept for safety-critical systems was validated using an experiment with 50 subjects that performed a computer-based C2 task while measuring valence and arousal.

We found an overall negative correlation between performance and arousal in most participants; thus, low arousal seems to be favorable to performance in almost all cases. For the valence dimension of emotion, however, we found both positive and negative correlations of performance with valence that varied interindividually; likewise, high interindividual differences in the subjects' general emotion were observed. These results prevent providing a generalizable recommendation toward promoting a particular type of emotion in a tutoring system for safety-critical work environments. It instead became apparent that affect-adaptive systems should consider individual emotional reactions.

A cluster analysis with correlation coefficients allowed categorizing subjects according to their similarities and differences in the relationship between emotion and performance. Three distinct clusters were identified and indicated the presence of a structure according to the silhouette index of 0.376. ARC 1 was clearly separable from the others, as subjects in cluster A showed negative correlations for both components of emotion. ARC 2 and ARC 3 merged into a single ARC with a weak positive correlation between valence and performance. ARC 0 had overall weaker correlations of arousal and performance than the other three clusters, and these correlations were not always negative as in the other groups. The correlations between valence and performance were also weaker and varied in direction. The results show that a categorization of users according to their emotion-performance relationship seems possible.

### Interpretation

**Figure 10:**
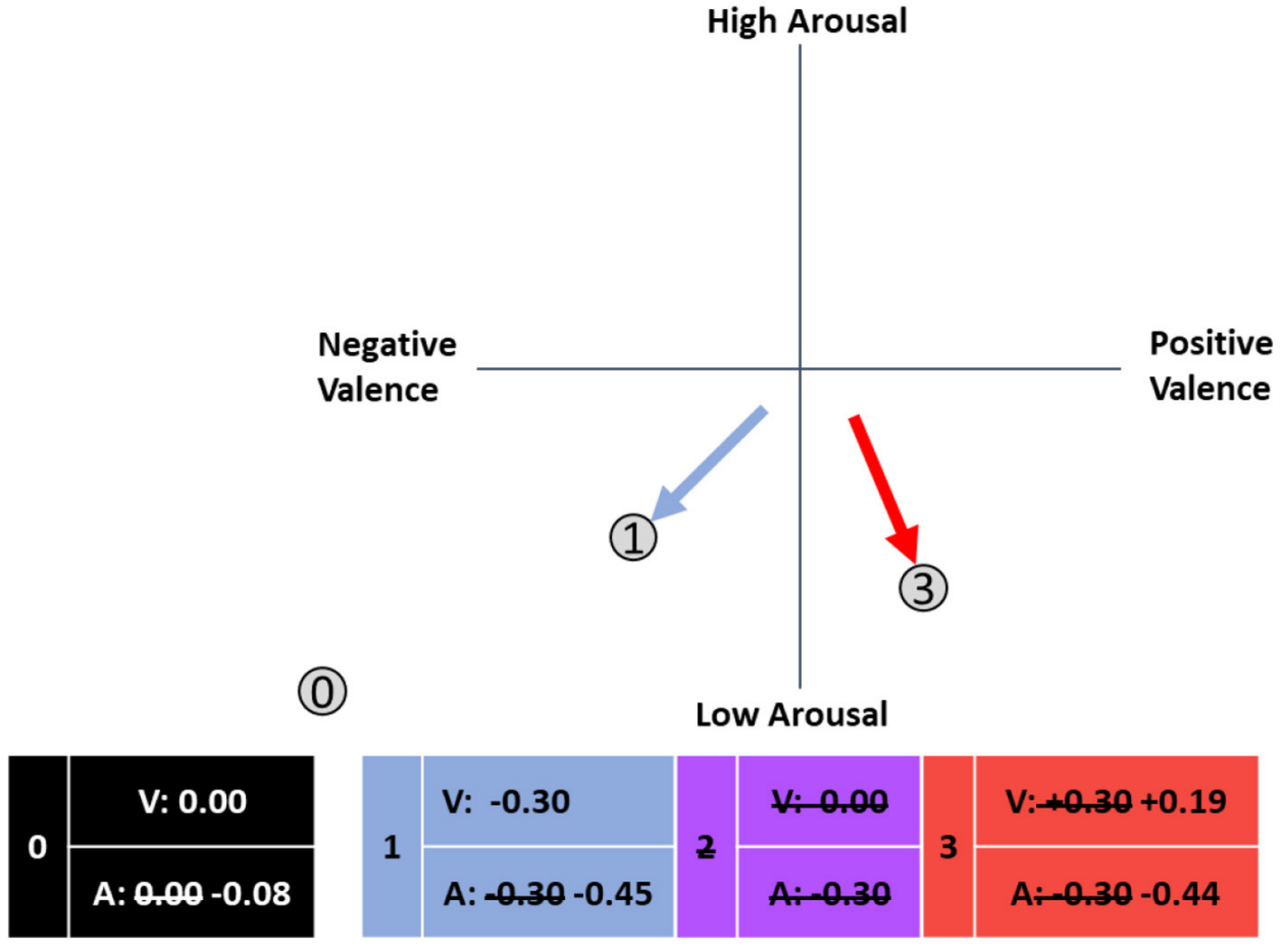
Adapted framework of affective response categories in the circumplex model of affect (adapted from Russell, 1980).

Based on the results of the cluster analysis, the originally established framework of relevant ARCs (Figure 3) required revision. An adapted version is shown in Figure 10. In addition to the numbered ARCs, their respective mean correlation coefficients for valence^*^performance (V) and arousal^*^performance (A) are shown. When the mean observed correlation coefficient varied significantly from the expected value, the mean original value is crossed out. When an ARC was not found, the number in the box is also crossed out.

- ARC 0 was clearly identified even if the mean correlation of arousal was lower than expected and the original framework required adjustment; nevertheless, the correlation coefficients for A^*^P and V^*^P do not reach the threshold for small effects (0.1) according to Cohen (1988). These results indicate that members of cluster B exhibit no emotion-performance correlations, and affect-adaptive mechanisms would probably not improve performance for these subjects.
- For ARC 1, we also found evidence in the data: while the correlation of performance and valence matched our expectations, the relationship with arousal was stronger than suspected and therefore corrected. Members of this ARC potentially benefit from a state of low arousal and negative valence, which could be induced and monitored by an affect-adaptive system using the proposed categorization system.
- We could not find evidence for ARC 2 as a single category, which is why the arrow was removed in the revised version. In the examined setting and task, no category of subjects is present that shows no link between valence and performance, but whose performance would benefit from low arousal.
- ARC 2 and ARC 3 apparently merged into one category that was assigned to ARC 3 but now has a smaller value for V^*^P. The new value indicates a small effect according to Cohen (1988) and therefore corresponds to ARC 3 instead of ARC 2. The arrow representing ARC 3 was shifted downwards to reflect the lower-than-expected correlation with positive valence, although the exact direction of the arrows does not perfectly correspond to the correlation as described in Section Methods. The arousal-performance relationship was negative but stronger than expected and was revised in the model. Subjects in this group would benefit from a state of positive valence and low arousal.
- As hypothesized in the original framework, no evidence could be found for ARCs 4 through 8, possibly due to the task simulating a safety-critical environment.

### Implications

The results of this contribution have theoretical implications and practical implications for the research field of ATS.

#### Theoretical Implications

Considering the present results, contradictory findings from the literature appear in a new light. For example, Malekzadeh et al. (2015) found that positive emotion was beneficial for learning performance. Members of ARC 3 were possibly predominant in their study since a positive valence-performance relationship is suspected in this category. The positive influence of negative emotions such as frustration or anxiety, as experienced by members of the ARC 1 category, reflects the work of Picard (2000). It is possible that ARCs were present in all of these studies but were not identified due to group-level analyses. Depending on sampling effects or task characteristics, one or another ARC may have dominated, influencing the global results of the respective study. In future studies of affect-adaptive systems, we suggest to review the ARC affiliation of study participants.

#### Practical Implications

**Figure 11:**
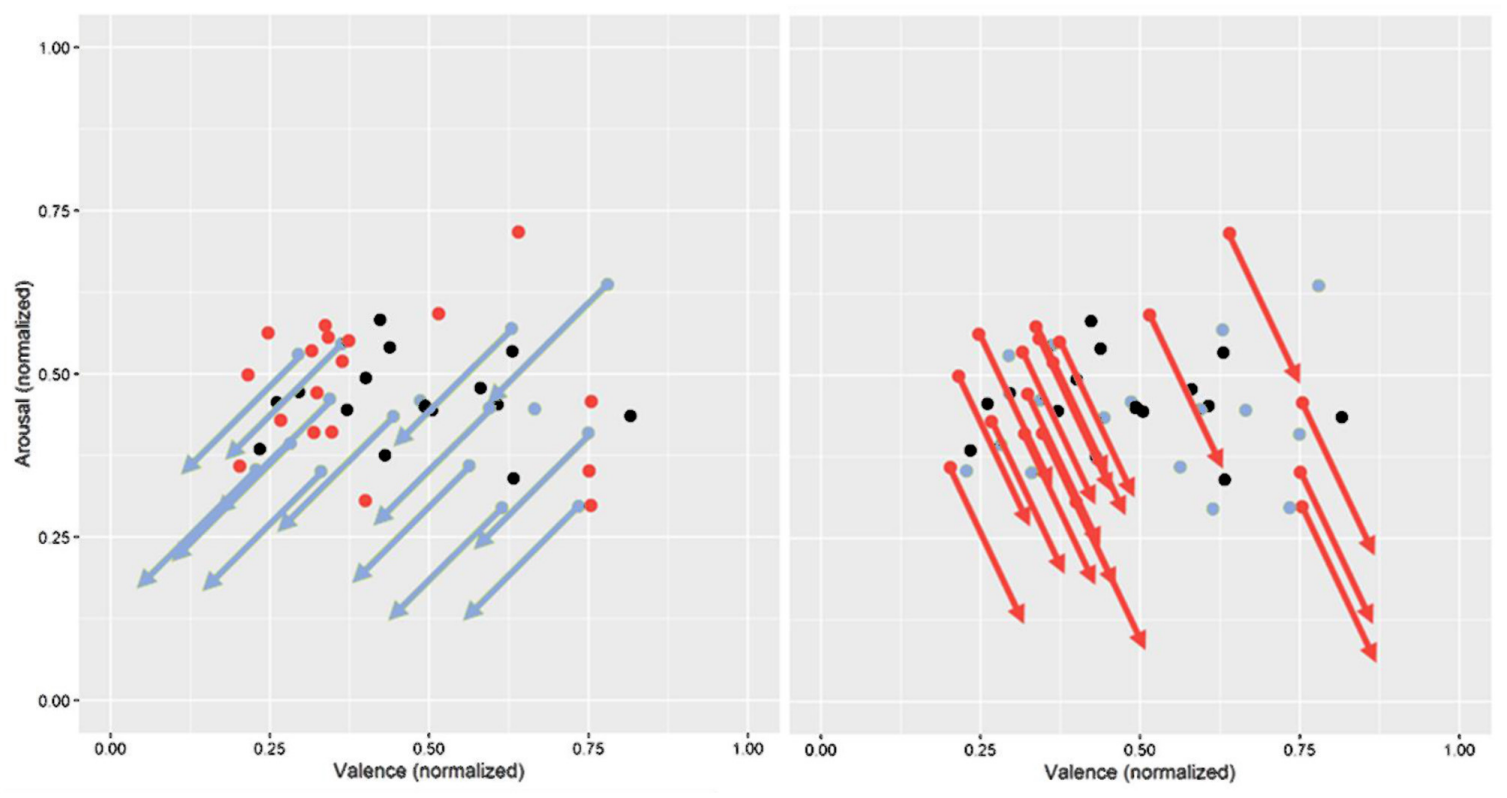
Application of the adaptation directions to the identified clusters (ARC 1: blue dots and arrows, ARC 3: red dots and arrows).

The ARC concept could be valuable to the concrete implementation of an ATS. The assignment of students to different categories allows considering individual emotional needs in task processing. This consideration was illustrated using the data of the present experiment. Figure 11 shows the mean values of emotion and valence of all subjects similarly to Figure 6 but are now color coded according to cluster membership, where members of ARC 1 appear as blue dots, ARC 3 as red dots, and ARC 0 as gray dots. On the left, blue arrows indicate the ideal adaptation directions for members from ARC 1 based on the ARC concept. Following the same principle, the ideal adaptation direction for ARC 3 is indicated in red on the left. Most members of ARC 3 had their general emotion located in the negative valence range, which might be important information for future work in assigning students to ARCs. The general emotion combined with knowledge of ARC affiliation will allow a real-time classification of the emotional state into critical and non-critical.

Further implications can be derived for designing affect-adaptive learning systems in safety-critical domains. One goal in this regard should be to maintain low arousal, since this state was associated with high performance for almost all subjects; for example, the color design might utilize cool, calming colors rather than warm colors that promote activation (Levy, 1984). A calming effect could also be achieved *via* proper lighting (Wessolowski et al., 2014). Anti-stress balls for kneading could also provide stress relief (Srivarsan et al., 2021) if the task can be performed with one hand. Furthermore, specific adaptive mechanisms are considerable, which we divide into the direct adaptation of the emotional user state and the adaptation of the interaction in the following.

A virtual tutor or pedagogical agent as described in Petrovica et al. (2017) can be imagined that could have a calming influence, which should tend to have a calming influence to reduce arousal and respond individually to the student depending on the ARC. Members of ARC 1 possibly have higher learning effectiveness to negative responses, such as criticism or pressure. Members of ARC 3 should perhaps receive more praise and appreciation to induce positive emotions; however, influencing valence without simultaneously increasing arousal might pose a challenge. A reward or sanction mechanism could have similar effects, where rewards in the presented task could include level-ups and monetary payoffs based on points achieved, while sanctions could be the loss of “game lives” or monetary loss of an initial achievable sum. Further exploration of the connections between emotional and motivational user states as described in Schwarz and Fuchs (2017) could be worthwhile in developing appropriate adaptation strategies.

Furthermore, recreational breaks in task processing can serve the purpose of inducing emotions. Interruptions could reduce arousal by guiding exercises such as shoulder circles, stretching, or meditation. Moreover, visual emotion inductions such as mini-games, pictures, or videos (Joseph et al., 2020) according to the ARC affiliation also represent an option during brief breaks. In addition, musical emotion induction (Västfjäll, 2001) is a promising approach to directly adapt the emotional user state. Emotion induction by music would only occupy the auditory sensory channel and thus interfere less with task processing compared to visual measures (Wickens, 2008). Västfjäll (2001) list pieces of music suitable for inducing positive, negative, and neutral valence in combination with low or high arousal, respectively. Knörzer et al. (2016) showed that negative emotions induced by musical pieces support learning, whereas induced positive emotions interfere with learning processes. For safety-critical environments, pieces inducing low arousal could be selected.

### Limitations

The interpretation of the present investigation and its results requires considering several limitations. In the present experiment, only correlative relationships were examined; no conclusions can be drawn about the causal direction of action. For the adaptive mechanisms in an affect-adaptive system, the direction is important; for example, it would make a difference whether negative emotions caused a decrement in performance or whether noticing low performance led to annoyance or frustration. Furthermore, we have not yet shown whether a user's affiliation to an ARC is consistent and stable over time across various difficulty levels, tutoring systems, or task environments. In the present study, the relationship between emotion and performance of each subject was analyzed across different scenarios to enable dividing the sample into ARCs and thereby assign subjects to them. It remains unclear whether this assignment is stable over time.

In the present study, major individual differences were found not only in the emotion-performance relationship but also in the average mood of the subjects. These findings are relevant for affect-adaptive systems because they provide the starting point for adaptation. A possible explanation for these differences is offered by appraisal theory, which states that emotions are evoked by evaluating a stimulus and its correspondence to individual goals and expectations. Several processes such as bodily sensations and situational factors contribute to the emotional experience (Moors, 2017). We hypothesize that individual differences in subjects' general emotions arise during the appraisal phase. For example, if a user tends to use an appraisal style that is prone to anger, events are often appraised in a way that leads to feelings of anger (Scherer et al., 2001), which is reflected by a tendency toward negative valence. Another explanation for the observed individual differences might be found in personality traits. Previous research suggests that the Big Five personality traits play an important role in this process (Tong et al., 2006); therefore, the assignment of users to ARCs may be enhanced by including personality traits as additional input.

In addition, methodological limitations must be considered in the present investigation; for example, the employed performance measure is based on the performance score described in Becker et al. (2021). This score considers correct and timely task processing and task priority but measures task performance rather than learning performance. The use of this performance measurement metric was necessary, however, to determine the optimal emotional performance state while initially interacting with the learning system. Another issue with the employed performance metric and the creation of the scenarios regarded the ceiling effects that occurred in this study. Two levels were sufficiently easy for ceiling effects to occur with the result that the affected scenarios had to be excluded. The resulting data loss could have been avoided by using more difficult scenarios with a higher task density. The scenarios were based on the WCT, which contains a large number of tracks occurring at the beginning leading to negative effects on the performance score. Furthermore, the descriptive analysis showed higher arousal in the more difficult levels. Since the performance was also lower in these scenarios, the correlation between high performance and low arousal could be partly due to the variance in difficulty levels and in task distribution within the scenarios. Further methodological limitations arise due to normalization of the individual data that renders interpretation of the general emotion more difficult. This procedure was still necessary as physiological data like pupil width is subject to interindividual deviations, for example due to age factors (Kasthurirangan and Glasser, 2006).

The employed FACET algorithm (see Section Methods) is based on the Computer Expression Recognition Toolbox (CERT; Littlewort et al., 2011), which was evaluated using the Extended Cohn-Kanade Dataset (Lucey et al., 2010). This dataset contains posed facial expressions differing in age and heritage. Results of the evaluation (for details see Littlewort et al., 2011) indicate a good recognition accuracy of 90.1%. Due to these results and the diversity of the test material, the current emotion recognition cannot be biased by the employed FACET algorithm. FACET was further validated against other out-of-the-box automatic classifiers for emotion recognition and was found to provide higher classification performance than other engines tested (Bernin et al., 2017; Stöckli et al., 2018; Dupré et al., 2020). Furthermore, the unified valence scale is based on the validated AffectNet database (Mollahosseini et al., 2017). The validation of the ARC concept, however, was conducted on a non-representative sample containing only employees of the FKIE. To ensure the third subprinciple of provable benefice regarding generalizability to an intended sample, future research needs to replicate the ARCs in a cross-cultural and more heterogeneous sample. Lastly, the fourth subprinciple outlines AI transparency and accountability as part of provable benefice. In Section Methods, the sample data, selected cluster algorithm and databases, and their associated limitations are described in detail. Future operators need to be aware of these limitations when using the training system in practice.

### Future Work

To investigate the direction of the emotion-performance relationship, an experiment that includes emotion induction, meaning the experimental manipulation of emotional state, would be helpful. Furthermore, other learning environments could be used that inherently lead to more positive mood than would be the case with an airspace surveillance task; Sykes (2005) for instance, developed an intelligent java tutoring system that 79% of students rated as enjoyable. A naturally enjoyable and fun tutoring system could possibly identify ARCs 4 through 8, which were not observed in the present study. Follow-up studies that consider the aforementioned limitations are needed to validly replicate the ARC concept.

Verifying whether the user's affiliation to an ARC is consistent and stable over time across various difficulty levels, tutoring systems, or task environments would require conducting a study design with measurements repeated over several days with the same subjects. If subject assignment to their respective ARCs proves to be consistent over several days, consistency over time can be assumed. If time stability cannot be proven, an operational affect-adaptive system would need to regularly establish a new ARC assignment for correct adaptation, for example *via* a daily baseline. By collecting individual user data regarding emotion and performance over time, systems may create an increasingly accurate personalized model of the emotion-performance relationship. Stability should also be ensured across varying difficulty levels so that performance can be enhanced depending on the current difficulty level. The correlation between emotion and performance is possibly stronger in more difficult settings, leading to a higher demand for affect-adaptive mechanisms in such scenarios.

An operational system should be able to assign users to ARCs shortly after system start, because adaptations that consider individual differences can only be employed after the assignment. Further research is necessary to develop an algorithm that enables fast and reliable allocation. A first approach could investigate similarities between individuals in the ARCs concerning personality traits, age, and gender. An additional approach could record baseline emotions and performance as a basis for the assignment. As the task progresses, the emotional state would be recorded in real time and the ARC could be used to assess whether it is located on the Circumplex Model in a range beneficial to performance (uncritical) or not (critical). If decrements in performance are detected, for example, a parallel classification of the emotional state could assist the system in selecting an appropriate adaptation in order to stabilize performance. This would fulfill the goal of ATS according to Petrovica et al. (2017), which is adaptation to the student's emotional state with the goal of intervening when an emotional state negatively impacts knowledge acquisition and learning outcomes. When the student is a member of ARC 0, adaptations targeting the emotional state may not be useful. This information is also important for an adaptive tutoring system, which can thus focus on adapting other states such as misdirected attention or high stress (Fuchs et al., 2020). According to Hudlicka (2003), however, appropriate monitoring procedures need to be implemented to prevent undesirable or dangerous extremes.

## Conclusions

This article describes the concept of ARCs for categorizing students in affect-adaptive learning systems in safety-critical systems based on their individual relationships regarding emotion and performance. The concept was developed from various preliminary studies and tested in a laboratory experiment. The results of a cluster analysis show that a subdivision of the learners into three groups is possible. One group shows high performance associated with negative valence and another with positive valence. This classification should be taken into account by an affect-adaptive system to select appropriate adaptive mechanisms to achieve the best possible performance by an individual. One-third of the subjects showed no connection between emotion and performance and thus would not benefit from affect-adaptive mechanisms. The present article contributes to the research field of affective computing by confirming the presence of interindividual differences in the relationship between emotion and performance and suggesting a validated concept that allows a future ATS to consider them.

## Data Availability Statement

The raw data supporting the conclusions of this article will be made available by the authors, without undue reservation.

## Ethics Statement

The studies involving human participants were reviewed and approved by Ethics Committee of the University of Technology Chemnitz. The patients/participants provided their written informed consent to participate in this study.

## Author Contributions

ASH organized and wrote most of article, developed, and validated the concept. SMS contributed to the user ethics section and data collection. RB developed the experimental testbed and provided technical support in conducting the experiment, data preparation, and analysis. SF organized funding, provided input to several scientific aspects, and improved clarity and intelligibility. MW gave valuable input to concept and statistical analyses and significantly improved structure. All authors contributed to the article and approved the submitted version.

## Funding

This research was funded by TALENTA, a scholarship program for women at Fraunhofer, and public grants.

## Conflict of Interest

The authors declare that the research was conducted in the absence of any commercial or financial relationships that could be construed as a potential conflict of interest.

## Publisher's Note

All claims expressed in this article are solely those of the authors and do not necessarily represent those of their affiliated organizations, or those of the publisher, the editors and the reviewers. Any product that may be evaluated in this article, or claim that may be made by its manufacturer, is not guaranteed or endorsed by the publisher.
